# Sialic Acid-Siglec Axis in Human Immune Regulation, Involvement in Autoimmunity and Cancer and Potential Therapeutic Treatments

**DOI:** 10.3390/ijms22115774

**Published:** 2021-05-28

**Authors:** Elena Gianchecchi, Andrea Arena, Alessandra Fierabracci

**Affiliations:** 1 VisMederi srl, Strada del Petriccio e Belriguardo, 35, 53100 Siena, Italy; elegianche@yahoo.it; 2 Infectivology and Clinical Trials Research Area, Bambino Gesù Children’s Hospital, IRCCS, Viale San Paolo 15, 00146 Rome, Italy; aarena026@gmail.com

**Keywords:** siglec, sialic acid, autoimmunity, cancer, treatment

## Abstract

Siglecs are sialic acid-binding immunoglobulin-like lectins. Most Siglecs function as transmembrane receptors mainly expressed on blood cells in a cell type-specific manner. They recognize and bind sialic acids in specific linkages on glycoproteins and glycolipids. Since Sia is a self-molecule, Siglecs play a role in innate immune responses by distinguishing molecules as self or non-self. Increasing evidence supports the involvement of Siglecs in immune signaling representing immune checkpoints able to regulate immune responses in inflammatory diseases as well as cancer. Although further studies are necessary to fully understand the involvement of Siglecs in pathological conditions as well as their interactions with other immune regulators, the development of therapeutic approaches that exploit these molecules represents a tremendous opportunity for future treatments of several human diseases, as demonstrated by their application in several clinical trials. In the present review, we discuss the involvement of Siglecs in the regulation of immune responses, with particular focus on autoimmunity and cancer and the chance to target the sialic acid-Siglec axis as novel treatment strategy.

## 1. Introduction: Biology of the Sialic Acid-Sialic Acid-Binding Immunoglobulin-Like Lectin (Siglec) Axis

The membrane of every living cell, in addition to nucleic acids, lipids, and proteins, displays various glycans (carbohydrates). Protein post-translational modifications with glycans profoundly influence their functions by modifying cell physical properties, shaping signal transduction and they have a part in cell-cell interactions. In addition, both secreted glycoproteins and glycosaminoglycans (GAGs) within the extracellular matrix (ECM) exert an impact on migratory patterns and interactions of immune cells. In addition, lipids also undergo changes through the binding to glycans, forming glycolipids. Glycans of cell surface and secreted glycoconjugates of mammalian cells are often terminated with sialic acids (Sias) to form sialoglycans [1,2]. All cell surface glycans constitute the glycome [3].

Methodological issues in studying glycans have been overcome only in the last decades with the availability of new technologies that have allowed to explore glycan complexity [4]. Even though glycans represent fundamental molecules for living organisms their role in physiological conditions as well as in disease has been only partially elucidated [1]. The diversity of the glycome greatly exceeds the genome and proteome diversity due to the possibility of different linkages involving these molecules [3]. Sias family comprises about 50 naturally-occurring negatively-charged carbohydrates sharing a nine-carbon neuraminic acid skeleton with a carboxylic acid at C1 and the anomeric center at C2 [5]. In the Golgi apparatus of mammals, 20 rather conserved enzymes defined sialyltransferases attach sialic acids via a diversity of linkages (α2,3, α2,6, and α2,8); this enables cells to display on the cell membrane a great variety of sialoglycans with great structural diversity that constitute the so-called ‘sialome’ [6,7,8]. Two most represented Sias are *N*-gycolyl-neuraminic acid (Neu5Gc) and *N*-acetyl-neuraminic acid (Neu5Ac) in the majority of mammalian systems [9]. Concerning humans, a mutation affecting the *CMAH* gene does not allow them to synthesize the Neu5Gc; however, it can be metabolically incorporated from external sources, such as red meat, playing a role in cancer progression and atherosclerosis through the involvement of the humoral inflammatory response [10,11,12].

**Figure 1:**
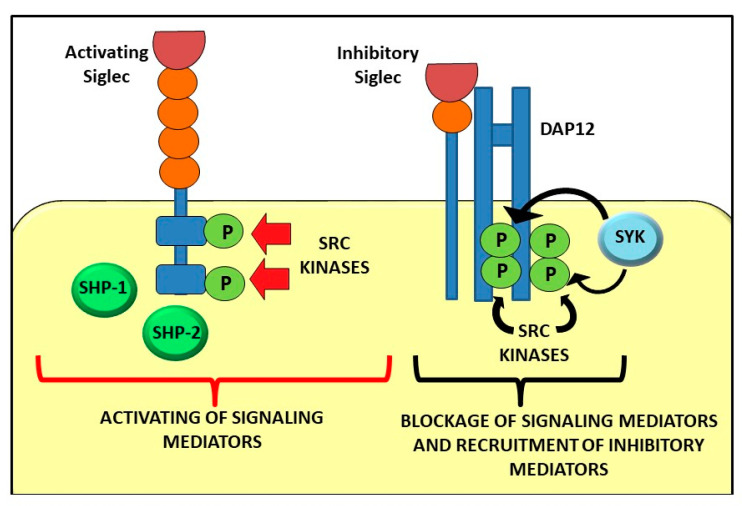
Signalling pathway mediated by activating and inhibitory Siglecs.

The structural diversity of sialoglycans explains the various functions of these sugars in human physiology (e.g., protein folding, neural development and cell–cell interactions), although many of their effects remain to be fully elucidated [1]. Sias function both at molecular and cellular level by interacting with selectins, factor H, and Sia-binding immunoglobulin (Ig)-like lectins (Siglecs). The term Siglec was firstly used in 1998 [2] to refer to a family of I-type lectins, immune regulatory receptors within the mammalian immune system that displayed binding preferences for Sia modifications [1]. Siglecs are single-pass type 1 transmembrane proteins. They display an extracellular N- terminal V-set Ig-like domain involved through the carbohydrate recognition domain (CRD) in extensive molecular interactions with sialoglycans and variable number of the so-called ‘C2-set’ Ig-like domains (1 to 16 C, the latter in the case of sialoadhesin). These Ig domains present elevated sequence similarity to the structure of the variable (V) and constant Ig domains [13]. The great majority of Siglecs possess immunoreceptor tyrosine-based inhibition motifs (ITIMs) in their intracellular portion that is phosphorylated by Src family kinases [9]. Phosphorylation produces high affinity docking sites for Src homology region 2 domain-containing phosphatase (SHP)-1 and SHP-2 involved in the dephosphorylation of nearby tyrosine-phosphorylated receptors and leading to the inhibition of downstream activation pathways (Figure 1) [14].

The modulation of intracellular signaling can occur for example through the inhibition of integrin-mediated signaling by Siglec-E in murine neutrophils [15]. Activating CD33-related Siglec receptors lack ITIMs, but they are characterized by a positively charged amino acid within the transmembrane domain implicated in the binding to DNAX-activating protein of 12 kDa (DAP12) [16]. The linkage between DAP12 and activating Siglecs leads to the phosphorylation of the immunoreceptor tyrosine-based activating motifs (ITAMs) of DAP12 and to spleen tyrosine kinase (Syk) activation [14,16]. Syk, in turn, can inhibit or activate downstream signaling depending on the cell type [17]. In addition, some human CD33-related Siglecs are paired with an activating receptor characterized by similar extracellular portions but different transmembrane and cytoplasmic domains, such as inhibitory Siglec-5 is paired with activating Siglec-14, or inhibitory Siglec-11 with activating Siglec-16. It has been hypothesized that the role of these paired receptors is to counteract the effect caused by the engagement of inhibitory Siglecs on immune cells by pathogens which would allow them to escape immune recognition [14].

Siglecs are immunomodulatory receptors most of which exert immunosuppressive functions (*vide infra*) analogously to other immune checkpoints inhibitors i.e., PD-1 and CTLA-4 [17,18]. During the past 30 years, 17 members of the Siglecs were discovered in hominoid primates with 14 Sia-binding members in humans. They are preferentially expressed on white blood cells of the immune system generally harbored by glycoproteins and glycolipids. Except for resting T cells, the majority of human and murine immune cell types show the expression of at least one Siglec [14]. Based on sequence similarity and evolutionary relatedness, these receptors are subdivided into two broad categories. The Siglecs of the conserved group include Siglec-1 (sialoadhesin, CD169), Siglec-2 (CD22), Siglec-4 (myelin-associated glycoprotein, MAG), and Siglec-15 in humans. Orthologues have been identified in all mammalian species investigated [14]. The genes encoding for these Siglecs are located on different chromosomes in humans even though characterized by a low (25–30%) sequence identity [16]. Instead, the second group of Siglecs, representing the main subset, are clustered on human chromosome 19, and refers to the CD33- (Siglec-3)- related Siglecs (CD33rSiglecs) (in humans they include Siglec-3, -5, -6, -7, -8, -9, -10, -11, -14, and -16) [9]. Inhibitory CD33rSiglecs able to bind a wide range of sialoglycans can be viewed as pattern recognition receptors able to discriminate endogenous sialoglycans as self-associated molecular patterns (SAMPs) preventing immune cell response in the vicinity of a sialoglycan-rich environment. Such a role has been demonstrated by the observations that halting the Sia–Siglec axis could even cause the breakage of peripheral tolerance and autoimmunity. A more restricted role could instead be played by those Siglecs characterized by a higher specificity for certain ligands [19]. Although this class of Siglecs shows ~50–99% sequence identity among the subfamily members, they are characterized by a rapid evolutionary diversification with significant differences in repertoire among mammalian species, especially between humans and the closely related great apes [14]. The number of genes encoding for Siglecs found to be correlated to the lifespan of mammals as well as the marked diversity of Siglec expression among mammals (with mice showing only 8 Siglecs with respect to humans with more than 15 Siglecs) suggest the evolutionary advantage of the acquisition of Siglecs in humans. In fact, Siglecs generated through multiple processes, such as gene duplication, but also conversion, deletion and exon shuffling and further diversification mechanisms including pseudogenization, gene conversion, altered expression and single amino acid substitutions that, in cases that occur within the CRD, lead to altered ligand binding, further increasing the diversity [20]. CD33rSiglecs do not always have clear orthologues in all mammalian species. Siglecs are involved in the establishment of immune tolerance conferring self-protection from autoimmune responses; at the same time the elevated diversity characterizing this subgroup derived by a selection pressure and interplay of pathogens which use this receptor system to modulate the Siglec axis for immune escape. Different relevant pathogenic microbes, including Group B streptococci (GBS) [21] and human immunodeficiency virus (HIV) [22], have exploited the Siglec–SAMP axis developing molecular mimicry. In more detail, they show sialoglycan-SAMP-like structures on their surface that dampen host immune responses via inhibitory Siglec engagement [23].

The number of genes encoding for Siglecs was found to be correlated to the lifespan of mammals as well as the marked diversity of Siglec expression among mammals (with mice showing only 8 Siglecs respect to humans with more than 15 Siglecs) suggesting the evolutionary advantage of the acquisition of Siglecs in humans. In fact, Siglecs are generated through multiple processes, such as gene duplication, but also conversion, deletion and exon shuffling and further diversification mechanisms including pseudogenization, gene conversion, amino acid substitutions and altered ligand binding [11]. Siglecs show distinct binding preferences for different Sia-terminated glycolipids and glycoproteins based on underlying sugars. Although sialylated ligands for different Siglecs are in most of the cases unique, sometimes they are non-specific. In greater detail, Sias constitute a group of sugars present on all mammalian cells as a mechanism for the discrimination between self and non-self [24]. The recognition of Sia by some Siglecs (Siglec-7, CD22 and Siglec-4) can be regioselective or, such as in the case of Siglec-9, multiple bindings can occur. Whereas the monomeric affinity showed by Siglecs for their Sia-containing ligands is quite weak (ranging from high micromolar to low millimolar) [25], all Siglecs are characterized by a conserved arginine residue in the Siglec V-set domain that plays a fundamental role in stabilizing the link through the formation of a salt bridge by interacting with the carboxyl group of the Sia [26]. So far, little is known about the dependency of Siglec binding to preferred natural sialoglycan ligands, whereas Siglec-1 prefers to bind α 2,3-linked sialylated glycans, α 2,6-linked sialylated ligands including N-acetylneuraminic acid, 2,6 galactosides (Neu5Ac, α(2–6) Gal) are uniquely bound by Siglec-2. Sialosides with α 2,3-linked SA and sulphated epitope are preferentially bound by Siglec-8 and Siglec-9 [27].

Siglec function is strictly related to the cell type and ligands they interact with, and their regulatory activities and subcellular localization are in relation to other receptors of the immune response. The expression of some Siglecs has been found on different cells, like Siglec-9 on neutrophils, monocytes, natural killer (NK) cells, and B lymphocytes. In contrast, only a few Siglecs are expressed preferably on one cell type, i.e., sialoadhesin (Siglec 1) on macrophages, CD22 on B lymphocytes and Siglec-8 on mast cells as well as eosinophils implicated in allergy reactions, although found expressed at low levels on other cellular elements. Siglec-15, characterized in 2007 and representing one of the most evolutionarily conserved Siglecs in vertebrates with 83% of identity in humans and mice, and phylogenetically distant from other family members, is specifically expressed on myeloid cells and osteoclasts. Usually it is not expressed on other immune cells and tissues [24]. In addition, different cell types can be characterized by the expression of more than one Siglec, such as Siglec-5, -7, -9 and -14 on neutrophils Siglec-3, -5, -7, -9,-10 on monocytes (Table 1) [28]. In addition, the finding of Siglec-10 and CD24 expression at the fetal-maternal interface has revealed their involvement in the mediation of immune tolerance [29]. Moreover, the expression and activity of Siglecs is not restricted to the immune system as demonstrated by the expression of Siglec-4 on cells of the central and peripheral nervous system and its role in axon maintenance and function [1] (Table 1).

Even though the majority of Siglecs work as receptors, some Siglecs can act as functional ligands, such as Siglec-1 [24]. Siglecs can also possess a microdomain localization relative to the microdomain localization of other cell activation complexes namely nanodomains or caveolae. An inhibitory Siglec and an activating receptor can be co-localized. Their co-localization can be antibody-mediated or strengthened by cis- or trans- ligand interactions. In more detail, with the exception of sialoadhesin whose extended structure is able to project its Sia-binding site away from the plasma membrane, limiting its cis interaction [30], biological functions of Siglecs are triggered principally by cis interaction. This is due to the fact that Siglec binding sites are usually ‘masked’ by cis interactions with other glycan ligands expressed on the same cell (Rev. in [14]). Among the effects exerted by the binding between a carboxyl group of sialylated glycoconjugates and a Siglec there are the reduction of the inflammatory response, phagocytosis inhibition and diminished cellular activation [27].

## 2. Sialic Acid-Siglec Interactions in Human Diseases

In greater detail, sialoglycans play important roles in pathological processes including infection [31,32], autoimmunity [33,34,35] and cancer (Table 1) [36,37,38], although many aspects remain unknown. Sia–Siglec interactions exert different functions in human physiology as they modulate the balance between recognition of self and non-self and mediate cell adhesion, cell signaling, and uptake of sialylated pathogens. Through these mechanisms and others they are able to modulate directly and not T lymphocyte activity. Consequently, abnormal Sia– Siglec interactions can contribute to the onset and development of diseases. Furthermore, even pathogens and cancer cells by expressing surface Sias can interact with Siglecs on immune cells to escape immune recognition. More specifically, some pathogens can use inhibitory Siglecs to dampen the immune response and benefit their survival [24]. Since most of Siglecs are expressed on immune cells (Table 1) the Sia–Siglec axis has attracted attention within the scientific community as a potential target for immune modulation in the prevention and treatment of several disorders, by using different therapeutic strategies (*vide infra*). In this review we analyzed the role of the sialic acid-Siglec interactions in immune mediated diseases and cancer and possible immunomodulatory therapeutic approaches based on Sia–Siglecs axis modulation.

### 2.1. Sialic Acid-Siglec Interactions in Immune-Mediated Diseases

Siglecs are nowadays recognized as playing an important role in immune regulation. Since Sias are present on all mammalian cells, Siglecs can help to discriminate self- and non-self and work as immunosuppressive checkpoint molecules to avoid undesired immune responses. Furthermore, their cell-specific expression and endocytic capacity make them suitable molecules for targeted drug delivery. Immune abnormalities may develop from altered synthesis or abnormal expression or binding of Siglecs and Sias in cis- or trans-interactions on various immunocytes where both are broadly represented [16]. Both genetic and environmental factors are responsible for the development of autoimmune disorders. Even if each autoimmune condition is linked to specific mechanisms for the development of the autoimmune response to self-antigen, several studies have demonstrated the involvement of the sialoglycan–Siglec axis in their onset.

More specifically, changes involving this pathway can cause abnormal reactions towards antigens as in allergies and also to the interruption of peripheral tolerance and autoimmunity. Siglec-3 signaling was involved in the modulation of activation and IgE signaling in mast cells. In greater detail, Siglec-3 recruitment could inhibit IgE-mediated anaphylaxis by partially reducing Syk phosphorylation and leading to a marked diminishment in phosphorylation of downstream kinases. In addition, Siglec-3 recruitment desensitized mast cells to allergen. Duan et al. reported that the tyrosine phosphatase Shp-1 played a role in Siglec-3/Siglec-3 ligand-mediated inhibition of mast cell degranulation [39]. However, it has been observed that genetic ablation of this Siglec exerted a limited effect on innate immune functions in myeloid cells of mice with a humanized immune system [40], its inhibitory role is strictly context dependent [39]. A reduced Sia ligand production and deficiency of Siglec-2 or Siglec-10 led to B cell iper-activation and the failure of inhibitory signaling involved in autoimmunity [41].

In addition to altered expression of Sia ligands or Siglecs, also the presence of genetic polymorphisms have been found to play a role in different immune-mediated diseases [42], such as Guillain-Barré syndrome [43], systemic sclerosis (SSc) [44], systemic lupus erythematosus (SLE) [45], asthma [46,47], chronic obstructive pulmonary disease (COPD) including also the frequency of COPD exacerbation (Table 1) [48]. Concerning Guillain-Barré syndrome, if common variants were not identified by genome wide association studies to be correlated with this disease, the recent study conducted by Alborzian Deh Sheikh [43] found two rare variants in Siglec-10, a Sia-recognizing inhibitory receptor present on B cells. The two variants, always present in association due to a marked linkage disequilibrium in subjects carrying them, encodes for R47Q and A108V substitutions in the ligand-binding domain and were considerably accumulated in patients affected by Guillain-Barré syndrome. However, recombinant Siglec-10 protein carrying only R47Q and not A108V presented a defective recognition and binding to gangliosides. This was a consequence of the significant change in the site of Siglec-10 involved in the binding of ligands. Such a finding supports the role played by the Siglec-10 variant in the development of this disease through the defective suppression of the production of antibodies recognizing gangliosides [43]. Concerning SLE, monocyte Siglec-14 expression was upregulated in patients with this autoimmune disease and correlated with lupus disease activity. Recently the group of Sajay-Asbaghi [47] investigated the possible correlation between six single nucleotide polymorphisms (SNPs) of Siglec-8, whose expression was limited on mast cells, eosinophils and basophils, and allergic asthma susceptibility in the Azeri population of Iran. The study reported a strong correlation between the disease and rs36498, hypothesizing that the latter could be able to influence Siglec-8 expression. The Siglec-9 rs2075803 G/rs2258983 A haplotype, corresponding to a Siglec-9 variant with a lower activity in suppressing inflammatory response, could represent a risk factor for the onset of emphysema [48].

Subjects showing Siglec-14 expression suffered frequent COPD exacerbations [49]. In accordance with this finding, Siglec-14 loss due to Siglec-14-null allele homozygosity correlated with a lower risk of COPD exacerbation in a Japanese cohort due to the attenuation of the inflammatory responses to non-typeable *Haemophilus influenzae* (NTHi) [49]. Siglec-5/14 polymorphism, which has been found to be expressed on amniotic epithelium, correlated with premature delivery in the case of GBS infection representing a neonatal pathogen that can come into contact with the fetus through placental membranes. This finding was correlated to Siglecs’ ability to modulate inflammatory responses of amniotic epithelium to GBS [50]. Concerning the role played by changes in Siglec expression in the development of autoimmune diseases, a reduced expression of Sia ligands for Siglecs -1 and 2 were discovered in patients affected by rheumatoid arthritis (RA), insulin-dependent diabetes (Type 1 diabetes, T1D), and autoimmune polyglandular syndrome [51,52]. Siglec-2 is expressed at low levels early in B cell development, it peaks on mature B cells and is engaged in signaling inhibition through the B cell receptor (BCR). It has been supposed that the modulation of BCR signaling pathway by Siglec-2 could be critically involved in the control of peripheral B cell tolerance as demonstrated by the observation that B lymphocytes from CD22-deficient mice presented an increase in BCR-induced Ca_2_^+^ signaling. This effect was due to the impossibility to recruit SHP-1, negative regulator of B cell signaling, to the BCR signaling complex [53]. The Siglec-1 molecule plays an important role in myeloid cell differentiation and is a relevant biomarker of RA being highly expressed on circulating monocytes of patients with respect to healthy adults [54]. Toll-like receptor (TLR) agonists enhanced Siglec-1 expression in circulating monocytes and tissue macrophages of SSc patients through type I interferon (IFN)-mediated activation [55]. In the peripheral blood of patients affected by SLE plasmacytoid dendritic cells associated with no expression of alpha interferon were discovered. Siglec-1 expression in peripheral blood has been recently discovered to be a biomarker of human SLE [56]. Sun et al. [57] identified 10 novel SLE risk variants and confirmed 20 known loci in individuals with Asian ancestry. Among the new variants, the most significant locus was GTF2IRD1-GTF2I at 7q11.23 (rs73366469), followed by DEF6, IL12B, TCF7, TERT, CD226, PCNXL3, RASGRP1, SYNGR1 and SIGLEC6. The new variants were able to alter gene expression in cis or in trans. In light of this, they investigated the presence of possible link between the new and known SLE loci and the putative molecular mechanisms responsible for SLE pathogenesis. The study highlighted the existence of both direct and indirect connections among them via gene regulation, protein and biochemical interactions. As a whole, considering the already known and new loci, the explained heritability of SLE increased to 24%. It has been hypothesized that additional variants may contribute to SLE pathogenesis through epigenetic regulation, rather than protein structure/function modifications. Among the 10 new variants, six correlated with other autoimmune diseases, such as celiac disease (CD), RA, T1D, and multiple sclerosis (MS), allowing to hypothesize pleiotropic effects [57]. Thornhill and colleagues [45] investigated the cell surface expression of Siglec-5/14, Siglec-9 and Siglec-10 on peripheral myeloid subsets and reported the upregulation of Siglec-14 on monocytes from patients affected by SLE. This correlated also with lupus disease activity evaluated both in terms of clinical and serological disease. In addition, SLE patient eosinophils were characterized by a meaningful reduction in Siglec-10 expression; however limited data about the function of Siglec-10 on this cell type are currently available even if it is known that Siglec-G, representing the mouse orthologue of Siglec-10, plays a role in B-cell tolerance [58]. Siglec genes have also been considered protective toward SLE development both in humans and mice [35]. In particular Siglec-12 could offer protection against SLE onset in Asian populations. Such a hypothesis would be confirmed by in vivo findings. In greater detail, two missense mutations in murine Siglec (highly homologous to human Siglec-12) were indeed identified in lupus-prone B6.NZMSle1/Sle2/Sle3 (Sle1–3) mice. The presence of such mutations was associated with marked autoantibody production, glomerular immune complex deposition and severe renal pathology in mice similar to human SLE nephropathy.

Several studies in mice and humans indicated that Siglec-2 and Siglec-10 are involved in B cell tolerance acting as immunosuppressive receptors that specifically counteract BCR signaling [59] and B cell proliferation in lymphoma [60]. As demonstrated by Jellusova et al. [61], Siglec-G and CD22 showed partly redundant functions in B cells playing a fundamental role in the maintenance of B cell tolerance regulating BCR signaling threshold. More specifically, whereas nether CD22-deficient nor Siglec-G-deficient mice on a pure C57BL/6 or BALB/c background, respectively, presented autoimmunity, CD22 x Siglec-G double-deficient mice presented increased B1 cell numbers and developed systemic autoimmunity [61]. In addition, aged Siglec-G x CD22 double-deficient mice spontaneously developed anti-DNA and antinuclear autoantibodies. As sustained by the hyperproliferative response of B lymphocytes upon stimulation with several TLR ligands, it is plausible that enhanced TLR responses might favor the development of the autoimmune phenotype. Furthermore, Siglec-10/G interaction with CD24 molecules may reduce inflammation and autoimmunity by halting immune activation in response to danger-associated molecular patterns (DAMPs) during tissue damage [62]. This interaction apparently also limits the severity of Graft Versus Host Disease (GVHD) [63]. Systemic autoimmunity was induced in mice by double deficiencies of both Siglec-G and FcrRIIb [64]. Effects on promoting or preventing origin of B cell lymphoma or autoimmunity may derive from Siglec-2, Siglec-G, Sia ligands and/or inhibition of their signaling pathway [65]. A recent study by Zhao [66] demonstrated the contribution of CD22 and CD72 in a murine scleroderma model reporting a marked reduction in skin and lung fibrosis severity in CD22^−/−^, CD72^−/−^, and CD22^−/−^/CD72^−/−^ mice in comparison to wild-type (WT) mice in the bleomycin-induced model. Similar results were observed also when mice were injected with hypochlorous acid, representing another experimental model of fibrosis. Furthermore, CD22^−/−^, CD72^−/−^, and CD22^−/−^/CD72^−/−^ mice showed a significant reduction of CD3+ T cells, CD8+ T cells, and F4/80+ macrophages infiltrating the skin than WT mice when treated with bleomycin.

Some Siglecs in addition to chronic autoimmune inflammation may be also involved in acute inflammation [67,68]. As regards an acute kidney injury model, Siglec-1-positive macrophages were involved in recruiting neutrophils [68]. Recent evidence suggests that Siglecs regulate inflammation by playing a membrane cross-talk with pattern recognition receptors (PRRs) upon exposure to pathogen-associated molecular pattern (PAMP) or DAMP antigens as shown for Siglec-3 [69]. Furthermore, soluble Siglec-5 could bind P-selectin glycoprotein ligand 1 (PSGL1) thus favoring leukocyte rolling and inflammation [70]. Heat shock protein 70 (Hsp70) as a ligand for Siglec-5 delivered an anti-inflammatory response, while inducing a proinflammatory signal when acting through Siglec-14. The functional consequences of this interaction in humans also rely on the presence of Siglec-14 polymorphisms [71].

Siglec-7 regulates pathways of apoptosis in human platelets through binding of gangliosides, without affecting activation, aggregation or cell morphology [67]. Siglec-7 is co-expressed with P-selectin on activated platelets [67]. Siglec-E suppresses neutrophil recruitment into murine lung tissues by inducing β2-integrin-dependent Nicotinamide adenine dinucleotide phosphate (NADPH) oxidase activation [72]. Moreover, Siglec-9 could lead to neutrophil apoptosis. This effect occurred through reactive oxygen species (ROS)- and caspase-dependent pathways depending on the proinflammatory cytokine milieu [73].

Control of inflammatory reactions also occurs through the involvement of certain ligands for Siglecs, i.e., Siglec-8 and Siglec-9 were upregulated upon the inflammation with NFkB in human airways [74]. In addition, exogenous dietary Neu5Gc may be incorporated into tissues (*vide infra)* thus be recognized by certain Siglecs such as Siglec-2 [75]. Thus incorporation of dietary Neu5Gc into human cell surfaces or exposed on commensal bacteria may generate neoantigens and specific autoantibody responses against it (*vide infra*) [76] leading to autoimmune-like chronic inflammation.

### 2.2. Sialic Acid-Siglec in Cancer

By contrast with protein biosynthesis, the process of glycan biosynthesis is not template bound, but generated by multiple interactions involving gene expression, substrate availability, cellular environment and the underlying structure of protein [17]. Data support the hypothesis that distinct glycosylation patterns guarantee advantages in terms of tumor growth, tumor dissemination, and immune escape. Such glycosylation modifications include also hyper- and xenosialylation [77]. The enhancement of sialoglycan density or hypersialylation represents a key feature of malignant transformation found in many cancer types and associated with their progression several decades ago [78,79]. Hypersialylation could promote the escape from immunosurveillance through an ‘enhanced self’ engaging several not mutually exclusive inhibitory pathways all based on protein–glycan interactions [77]. Cancer hypersialylation is linked not only to several pathways such as upregulation of sialyltransferases by oncogenes including Ras and c-Myc [80], but also to other factors as the excess of substrates and upregulation of Sia transport systems [77]. Indeed a few studies demonstrated that interactions with Sia-binding receptors (Table 1), including selectins, could significantly affect tumorigenesis and tumor progression. This effect occurred by changing the physical properties of cancer cells [81], enhancing capabilities of cancer elements to evade apoptosis [36] and influencing sialoglycans clustering in lipid rafts [82,83]. Further hypersialylation enhanced immune evasion through the interference with factor H, inhibition of the complement system [84] and the engagement of immunoregulatory Siglec receptors [85,86,87,88]. Tumor hypersialylation may also depend on the overabundance of substrates, increased branching of glycan, upregulation of sias transports and accumulation of their acceptor molecules [77]. Various studies document the xenosialylation [75], which is the presence of the “xenoglycan” Neu5Gc on the cell surface glycans of several human cancer types, including melanoma, retinoblastoma, colon cancer and breast cancer [89]. This represents a common observed phenomena critical for cancer progression [11], representing a possible target for immunotherapy. Even though the precise mechanisms responsible for the accumulation of glycans containing Neu5Gc in cancer has not been clarified, it has been hypothesized that it could be related to a general enhancement in Sia metabolism [90]. Neu5Gc, ingested with the diet, can be incorporated into tissues in trace amounts, in particular in tissues characterized by rapid growth and/or turnover rates, such as epithelia, endothelia, fetal tissues, and carcinomas. As reported by Bergfeld [91] Neu5Gc is converted to GalNGc and can then be incorporated into the glycosaminoglycan chondroitin sulfate, a component of skeletal bone and extra-cellular matrices. A consequence of xenosialylation is the presence in humans of various levels of autoreactive antibodies recognizing the lost sialic acid, representing a “xeno-autoantigen” that can lead to “xenosialitis,” an inflammatory state due to reaction against a xeno-Sia included in “self” molecules [92]. Such an inflammatory condition is considered to be pro-tumorigenic supporting the findings that an enhanced red meat intake is associated with high tumor risk [93]. The accumulation of Neu5Gc respect to Neu5Ac might alter the binding properties to Siglec receptors, as demonstrated by the highest affinity to Neu5Gc characterizing some of them [94].

Besides hypersialylation and xenosialylation, additional changes involving sialic acids were reported in cancer, although they would need a deeper knowledge, i.e., C5-hydroxyl modification of sialic acid, leading to 2-keto-3-deoxy-D-glycerol-D-galacto-nononic acid (KDN) [95]. The free form of KDN was reported in ovarian cancer [96] and in carcinoma of the head and neck [97]. In addition, some cancers present altered O-acetylation of sialic acid and in particular 9-O-acetylation [98,99].

Among the mechanisms used by cancer cells for immune escape, a higher expression of Siglec ligands has been reported in several types of tumors, such as Siglec-6 in human colorectal cancer-associated mast cells [100], Siglec-9 on tumor-infiltrating T cells from non-small cell lung cancer (NSCLC), colorectal, and ovarian cancer patients [101] and Siglec-15 in a broad spectrum of human cancers. Siglec-15 is considerably overexpressed in the bladder, kidney, lung and liver cancers even though the highest expression was revealed in colon cancer, endometrioid tumor and thyroid cancer [102]. Altered expression profiling of immune inhibitory Siglecs and their ligands were recently found also in patients with glioma [103].

The recent study by Rodriguez [104] identified monocyte-derived macrophages, whose differentiation was induced via signaling through Siglec-7 and Siglec-9 as a consequence of the enhanced sialylation, as contributors to the poor clinical outcome in pancreatic ductal adenocarcinoma (PDAC) tumor. Moreover, Sias can target different myeloid cells, as demonstrated by the diminishment in inflammatory response induced by Siglec-9 and the upregulation of programmed death ligand-1 (PD-L1) and IL-10 expression [104].

The presence of a certain degree of variability observed in the expression of Siglec ligands in cancers of different tissue-specific origin is implicated in the different immune escape strategies and thus influences the possible therapeutic approaches relying on Siglecs or their ligands [105]. The serum protein N-glycosylation signatures observed recently in neuroblastoma patients could potentially represent disease biomarkers [106]. In addition, aberrant glycosylation might constitute a possible marker to identify those patients resistant to standard chemotherapy as observed recently by Zahradnikova [107] who revealed an association between N-glycome modifications and resistance to platinum-based chemotherapy in ovarian cancer. The use of changes in N-glycosylation of proteins in tissue and serum samples as diagnostic and prognostic factors for the estimation of sensitivity to chemotherapy might be beneficial for these patients wherein alternative treatment strategies to standard chemotherapy are available [107].

## 3. Therapeutic Approaches Based on Siglecs

In view of the correlation between altered Sia–Siglec interactions and a rising number of pathological conditions including infection, autoimmunity, and tumor, therapeutic strategies acting on the Sia-Siglec axis have attracted the attention of researchers. Novel therapeutic approaches that utilize the properties of binding selectively carbohydrates of this family of lectins are currently being exploited in addition to ongoing measures of native, conjugated or engineered antibodies (Table 2). In addition, starting from natural sialosides, technological progress has allowed Sia mimetics (SAMs) to be formulated through single or multiple chemical changes at different carbons of the Sia backbone. SAMs are characterized by a higher affinity as well as specificity for Siglecs respect to natural sialic acid ligands and can be linked to nanoparticles, polymers, or surface glycoproteins and glycolipids of living cells.

Biodegradable drugs are novel platforms that under physiological conditions allow multivalent, high-avidity Siglec-specific carbohydrate–ligand interactions. An increasing body of experimental evidences suggests that antibody-based or glyco-targeting strategies by crosslinking or blocking the ligand binding site might induce or inhibit Siglec signaling in immune cells. Agonistic and antagonistic Siglec-targeting drugs can, therefore, be developed for suppression or activation of defined immune subsets based on the type of receptor being expressed [86]. In view of the pro-inflammatory function played by certain Siglecs, such as Siglec-2 [66], they could represent eligible therapeutic targets for the treatment of fibrotic pathologies, including SSc. A recent study conducted by Sun and colleagues [24] identified Siglec-15 as an immune suppressor with a relevant role in the up-regulation of different cancer types. This could represent a putative target for cancer immunotherapy in light of its elevated expression on macrophages and cancer cells. The mutually exclusive expression with PD-L1, allowed to hypothesize that Siglec-15 could constitute an important immune evasion mechanism in PD-L1-negative patients. In more detail, anti-PD-1/PD-L1-resistant patients could benefit of Siglec-15 targeting therapies [24].

The identification of a correlation between immune-mediated conditions and Siglec expression or certain polymorphisms involving Siglecs as well as their downstream signaling pathway could be therapeutically relevant through the stratification of patient populations for personalized management, with a more intensive monitoring or an earlier medical intervention in order to limit the progression of the disease in patients with genetic variants that constitute risk factors for frequent disease exacerbation [42]. This could be particularly important in ethnic groups wherein the expression of specific Siglecs or polymorphisms is higher than in other geographical areas. In more detail, the higher Siglec-14 frequency expression observed in Africans and Europeans predispose such populations to a higher risk of COPD exacerbation than Asians characterized by a higher frequency of Siglec-14 null allele [108]. In addition to the use as target to drive immune responses, Siglecs can constitute also predictive biomarkers for the efficacy of cancer immunotherapy, as it has been recently demonstrated by Yamada [109].

### 3.1. Therapeutic Targeting of Siglecs Using Antibody-Based Approaches

Siglecs are considered attractive therapeutics targets in a wide range of diseases including autoimmune disorders and cancer. Since of their cell type-specific expression pattern and endocytic properties, antibodies targeting Siglecs could act as potential carriers for drug delivery (Table 2). Antibody-based therapies targeting Siglecs are under development representing promising therapeutic approaches. More specifically, they include unconjugated antibodies, antibody–drug/toxins, bispecific antibodies and chimeric antigen receptors (CAR) (Table 2). As recently demonstrated by Schanin et al. [110] the use of a monoclonal antibody to Siglec-8 (anti-S8) was able to halt non-allergic airway inflammation and inhibit IgE-independent mast cell activation in two in vivo models [110]. In more detail, the investigation of mast cell transcriptomic profile highlighted that anti-S8 was able to downregulate the pathways promoted by IL-33, including tumor necrosis factor (TNF) signaling via NF-κB supporting the use anti-S8 mAb in both allergic and non-allergic inflammatory diseases wherein mast cells are involved [110]. It is well known that the enhanced sialylation characterizing cancer cells can inhibit anti-tumor responses leading to tumor evasion from immune surveillance. Since this effect occurs through the involvement of Siglec-7 and Siglec-9 on NK cells, myeloid cells or T cells, preclinical studies based on the block of Siglec-7 or Siglec-9 with antibodies are ongoing (Rev. in [24]). Of note several antibodies against Siglecs are presently evaluated in clinical trials. Epratuzumab is a humanized unconjugated monoclonal antibody against Siglec-2 currently in two Phase III clinical trials in patients with SLE. The mechanism of action of Epratuzumab involves the immunomodulation of B cell signaling by inducing loss of BCR-related proteins [111] and inhibiting the BCR-signaling [112]. Lirentelimab (AK002), a humanized non-fucosylated immunoglobulin G1 (IgG1) monoclonal antibody directed against Siglec-8, is currently in Phase 2/3 clinical trials to evaluate its efficacy and safety in adult and adolescent patients with active eosinophilic esophagitis [113]. Alemtuzumab is a humanized monoclonal antibody that targets the Siglec-10, expressed at high levels on the surface of B and T lymphocytes. In 2001, Alemtuzumab was approved in the United States and Europe for use in patients with chronic lymphocytic leukemia (CLL) [114]. Treatment with alemtuzumab leads to the depletion of circulating lymphocytes and in recent clinical trials in patients with MS a clinical benefit superior to interferon-β was demonstrated. NC318 is a monoclonal antibody targeting Siglec-15, which is expressed on macrophages and on multiple tumor cell types. In preclinical studies, Siglec-15 suppressed T cell proliferation and negatively regulated T cell function. NC318 blocked Siglec-15-mediated immune suppression and restored T cell function in vitro exerting anti-tumor immunity [24]. NC318 is currently in phase I/II clinical trials in patients with advanced or metastatic solid tumors [115].

Due to the high expression of Siglec-2 on B cell lymphomas several compounds such as inotuzumab ozogamicin are currently in Phase II clinical trials for the treatment of B cell acute lymphoblastic leukemia (B-ALL) [116]. Inotuzumab ozogamicin is an antibody-drug conjugate comprised of cytotoxic antibiotic N-acetyl-gamma-calicheamicin dimethylhydrazide (a DNA-binding cytotoxic agent) attached to a recombinant humanized monoclonal antibody targeting Siglec-2 [117].

Mylotarg (gemtuzumab ozogamicin), as inotuzumab ozogamicin, is an antibody-calicheamicin conjugate to Siglec-3. Mylotarg was initially approved by the US Food and Drug Administration (FDA) in 2000 for the treatment of acute myeloid leukaemia (AML) but 10 years later Pfizer voluntarily withdrew the treatment from the market because the confirmatory Phase III trial did not show a clinical benefit of adding mylotarg to the standard chemotherapy for AML [118,119].

Blinatumomab was the first bispecific antibody approved in the USA for the treatment of ALL: one arm of this antibody binds CD19, while the other arm binds CD3. Blinatumomab can potentiate unstimulated T cells and induce direct cytotoxicity against CD19+ cells [120]. In this way an increase in targeting specificity can be obtained by the use of bispecific antibodies respect to a therapeutic approach based on a single targeting. So far, various approaches relying on bispecific antibodies have been developed.

The bispecific antibody DT2219 recognizing at the same time Siglec-2 and CD19 was designed for the treatment of B-ALL [121]. DT2219 is a bispecific toxin that consists of the catalytic domain of diphtheria toxin (DT) and single-chain variable fragments (scFV) of antibodies targeting human CD19 and CD22.

Novel technologies, such as CAR T cell engineering approaches, may open future perspectives to the development of therapeutic molecules targeting Siglecs. CARs are chimeric recombinant membrane proteins that consist of three parts: an extracellular antigen recognition domain of the single-chain fragment variant (scFv) derived from an antibody), a transmembrane domain and an intracellular T-cell activation domain of CD3ζ. Following antigen recognition, CAR endodomains transmit activation as well as costimulatory signals to T cells leading to their activation [122]. CARs targeting Siglec-2 and Siglec-3 have been produced and are in early-phase clinical trials for the treatment of B-ALL and AML [123,124,125].

### 3.2. Therapeutic Targeting of Siglecs Using Glycan-Based Approaches

In recent years, applications for therapeutic targeting of Siglecs by using SAMs have been proposed as an alternative immune modulatory approach to antibody-based strategy. The discovery of high-affinity Siglec ligands was the starting point to the development of synthetic sialoside analogs obtained through chemical modifications of the natural Sia ligands that increases binding affinities and selectivity towards Siglecs. Compared with antibody-based approaches, the use of glycan ligands may offer advantages, such as lack of auxiliary function, low immunogenicity and reduced side effects. These biodegradable nanoparticles (NPs) often comprise drugs, toxin or siRNA encapsulated in a polylactic co-glycolic acid (PLGA) or polyethylene glycol (PEG) shell or liposomes as potential methods for targeted drug delivery. SAM-decorated NPs have been developed showing promising results in two mouse models of generalized sepsis and one of pulmonary injury. Concerning the therapeutic application of NPs in human diseases, the effectiveness of NP exploitation in human macrophages and in a sophisticated ex vivo model of human lung edema represent even more interesting data [126]. The benefit represented by the use of NPs derives not simply from their function as a cargo delivery mechanism to the Siglec-expressing cells, but also by the fact that SAM-decorated NPs can be employed also to trigger Siglec signaling [127]. In addition, they can be used in combination with other therapies, such as photodynamic therapy, radiotherapy and chemotherapy, amplifying their therapeutic effect [128]. Paulson and coworkers developed a Siglec-2 ligand-decorated liposomal formulation loaded with doxorubicin that actively targeted Siglec-2-expressing B cell lymphoma [62]. In addition, the group of Paulson developed a high-affinity and selective ligand for Siglec-1 (TCCNeu5Ac) which when displayed on liposomal NPs could efficiently target the Siglec-1 positive macrophages [129]. Due the restricted expression on primary AML cells and B-cell lymphomas, Siglec-3 and Siglec-2 have received considerable attention as pharmaceutical targets. For this purpose Rillahan et al. [130] using glycan microarrays and chemo-enzymatic strategy synthesized two compounds that selectively target Siglec-2 and Siglec-3 when conjugated to liposomal NPs. These examples demonstrate that SAMs-decorated NPs targeting Siglecs exerted immunomodulatory properties and could be applied in the treatment of a wide range of diseases including cancer and autoimmune disorders. The translation of nanomedicine to clinical practice requires, however, overcoming some challenges that include the restricted understanding of immune network during tumorigenesis and the heterogeneity of different tumors and individuals. At least, further limits are represented by their putative immunogenicity representing themselves potential antigenic structures, minimizing NP efficacy and even leading to severe complications. In addition, further data regarding their potential toxicity are necessary [131].

## 4. Conclusions

Due to their restricted expression pattern on the surface of immune cells, capacity to regulate receptor signaling and elevated expression in case of specific tumors, Siglecs represent attractive therapeutic targets for lymphoma/leukemia as well as autoimmune diseases by the use of antibody- and glycan-based approaches. Nonetheless, increasing knowledge of Siglec structure and the identification of key molecular determinants can help the design of synthetic glycan ligands targeting Siglecs and, at the same time, increase their selectivity and affinity to Siglecs. Furthermore, a deeper understanding of the functions of Siglecs in the tumor microenvironment could lead to new immunotherapeutic strategies based on the nature of Siglecs as endocytic receptors which could be utilized as a drug-delivery system carrying anti-cancer molecules to tumor cells [132].

## Figures and Tables

**Table 1:** Sialic acid-binding immunoglobulin-like lectin (Siglec) expression, biological function and correlation with pathological condition.

Siglec	Expression	Suggested Biological Function	Pathological Condition
Sig-1 (CD169)	Macrophages, Monocytes	Myeloid cell differentiation, antigen presentation, host defense	RA, systemic sclerosis (SSc), systemic lupus erythematosus (SLE), Group B streptococci, (GBS) defense, HIV permissive infection
Sig-2 (CD22)	B cells	B cell differentiation and tolerance	B-cell lymphomas, SSc
Sig-3 (CD33)	Myeloid progenitors, Macrophages, Monocytes, DCs, Microglia, Granulocytes	Myeloid differentiation progenitors, Regulation of inflammatory response upon Pathogen Associated Molecular Patterns (PAMP) or Damage- Associated Molecular Patterns (DAMP) antigen exposure	Leukemia, degeneration
Sig-4	Myelin of nerves	Maintenance of myelinated axons, Suppression of axonal regeneration after injury	Latent infection, Neuron degeneration
Sig-5	Neutrophils, Monocytes	Recognition and internalization of sialylated pathogens, Inhibition of immune cell activation (Co-paired with Siglec-14)	Prematurity, chronic obstructive pulmonary disease (COPD)
Sig-6	Trophoblasts, Mast cells, Intestine	Regulation of trophoblast proliferation and invasiveness, Inflammation	Preeclampsia, Allergy
Sig-7	NK cells, Neutrophils, Monocytes, Mast cells, Platelets	Regulation of pathways of apoptosis in human platelets, Immunosuppression IgE-mediated	Tumor evasion, Allergy, HIV infection
Sig-8	Eosinophils, Mast cells, Basophils	Induction of apoptosis in eosinophils	Allergic asthma
Sig-9	Neutrophils, Monocytes, DCs, NK and B cells	Inhibition of NK cell and neutrophil activation and function, Immune modulation of myeloid cells; Induction of neutrophil apoptosis, Infections, Checkpoint blocker, Modulation of the tumor immunological microenvironment	Sepsis, cancer progression, COPD, Allergy
Sig-10	B cells, DCs, NKs	Immune tolerance	Tumor Immunity, Graft Versus Host Disease (GVHD), Safe pregnancy
Sig-11	B cells, Macrophages, Microglia, Ovary stroma	Immunosuppression	Ovary cancer, Neuroprotection
Sig-12	Macrophages	Unknown	Hypertension treatment outcome
Sig-14	Neutrophils, Monocytes	Activation of proinflammatory pathway in monocytes, Recognition of sialylated pathogens	COPD, Prematurity
Sig-15	Osteoclasts, Macrophages	Regulation of osteoclast differentiation and bone resorption, Immune modulation of macrophages	Osteoporosis, Cancer
Sig-16	Microglia	*E. coli* defense, Neuroprotection	*E. coli* defense, Neuroprotection

**Table 2:** Therapeutic approaches based on Siglecs.

Human Siglec Target	Application
Sig-2 (CD22)	Epratuzumab (anti-CD22) for Sjögren’s syndrome, B cell leukemia and SLE; Inotuzumab ozogamicin (anti-CD22 monoclonal antibody (MoAb) conjugated with a toxin (calicheamicin)); DT2219 and chimeric antigen receptors (CARs) for the treatment of B cell acute lymphoblastic leukemia (B-ALL); Moxetumomab pasudotox (LumoxitiTM) for hairy cell leukemia; CD22 binding peptide (PV3) for malignant B cells; CARs in B-cell acute lymphoblastic leukemia (BCP-ALL)
Sig-3 (CD33)	CARs for the treatment of AML; blinatumomab for B-cell acute lymphoblastic leukemia (ALL); Anti-CD33 (Siglec-3) BI 836,858 ( MoAb) for acute myeloid leukemia (AML), myelodysplasia syndrome (MDS); Anti-CD33 lintuzumab (HuM195) (MoAb) for AML; CD16/IL-15/CD33 Tri-Specific Killer Engagers (TriKEs) (Combined peptides) for AML, MDS, mast cell leukemia; gentuzumab ozogamicin (mylotarg) (MoAb) for newly diagnosed and relapsed AML patients; Anti-CD33/CD3 BiTE (AMG330, Amgen) for AML; JNJ-67571244 for not responding AML patients at high risk of myelodysplastic syndrome
Sig-7	Ganglioside GD3 expression on target cells can modulate NK cell cytotoxicity via a Siglec-7-dependent mechanisms
Sig-8	Lirentelimab (AK002) (MoAb) for active eosinophilic esophagitis and Chronic urticarial; Monoclonal antibody towards Siglec-8 (anti-S8) halted non-allergic airway inflammation and inhibited IgE-independent mast cell activation in two in vivo models
Sig-9	[68Ga]-DOTA-Siglec-9 (radioisotope-peptide imaging) for RA
Sig-10	Alemtuzumab for CLL and MS
Sig-11	PolySia avDP20 reduced vascular leakage of laser injury in humanized transgenic mice expressing Siglec-11
Sig-15	Anti-Siglec-15 NC318 (MoAb) in patient with advanced or metastatic solid tumors

## Data Availability

Not applicable.
